# Stem Cell Therapy in Heart Diseases: A Review of Selected New Perspectives, Practical Considerations and Clinical Applications

**DOI:** 10.2174/157340311798220502

**Published:** 2011-08

**Authors:** Eltyeb Abdelwahid, Tomasz Siminiak, Luiz César Guarita-Souza, Katherine Athayde Teixeira de Carvalho, Pasquale Gallo, Winston Shim, Gianluigi Condorelli

**Affiliations:** 1 CBRC, Massachusetts General Hospital/Harvard Medical School, Building 149, 13^th^ Street, Charlestown Massachusetts, 02129, U.S.A; 2 Pozna University of Medical Sciences, Cardiac and Rehabilitation Hospital ul. Sanatoryjna 34 64-600 Kowanówko k/Obornik Wlkp. Poland; 3 PUCPR - Experimental Laboratory of Cell Culture Institute of Biological and Health Sciences, CCBS, Brazil; 4 Pequeno Príncipe Faculty & The Pelé Pequeno Príncipe Institute- Child and Adolescent Health Research, Curitiba,Brazil; 5 Laboratory of Molecular Cardiology, San Raffaele Biomedical Science Foundation of Rome, IT; 6 IRCCS, Multimedica Hospital, Milan, IT; 7 National Heart Centre Singapore, Singapore; 8 Department of Medicine, University of California, San Diego, CA 92003 USA,

**Keywords:** Heart, stem cells, transplantation, therapy.

## Abstract

Degeneration of cardiac tissues is considered a major cause of mortality in the western world and is expected to be a greater problem in the forthcoming decades. Cardiac damage is associated with dysfunction and irreversible loss of cardiomyocytes. Stem cell therapy for ischemic heart failure is very promising approach in cardiovascular medicine. Initial trials have indicated the ability of cardiomyocytes to regenerate after myocardial injury. These preliminary trials aim to translate cardiac regeneration strategies into clinical practice. In spite of advances, current therapeutic strategies to ischemic heart failure remain very limited. Moreover, major obstacles still need to be solved before stem cell therapy can be fully applied. This review addresses the current state of research and experimental data regarding embryonic stem cells (ESCs), myoblast transplantation, histological and functional analysis of transplantation of co-cultured myoblasts and mesenchymal stem cells, as well as comparison between mononuclear and mesenchymal stem cells in a model of myocardium infarction. We also discuss how research with stem cell transplantation could translate to improvement of cardiac function.

## HUMAN EMBRYONIC STEM CELLS AS A SOURCE OF CARDIOMYOCYTES FOR CELL THERAPY APPLICATIONS: OBSTACLES TO OVERCOME

In contrast to adult stem cells, embryonic stem cells (ESCs) have the potential to differentiate into tissue derivatives of all three embryonic germ layers and therefore are termed pluripotent. Cardiomyocytes (CMCs) have been obtained by all three types of murine embryo-derived stem cells: embryonic carcinoma (EC), embryonic stem (ES), and embryonic germ (EG) cells. In this chapter, we focus our attention on human ESCs (hESCs), due to their potential clinical application. hESC lines, isolated from the inner cell mass (ICM) of embryos can be propagated continuously in the undifferentiated state when grown on top of a mouse embryonic fibroblast (MEF) feeder layer. When removed from these conditions and grown in suspension, they begin to generate three-dimensional differentiating cell aggregates termed embryoid bodies (EBs). This *in vitro* differentiating system can be used to generate a plurality of tissue types. The ability of hESCs to differentiate into mature somatic cells was demonstrated using spontaneous and directed *in vitro* differentiation systems. So far, hESCs have been shown to differentiate into neuronal tissue [1], ß islet pancreatic cells [2], hematopoietic progenitors [3], endothelial cells [4] and cardiac tissue [5,6]. Interesting data were obtained by the use of adult stem cell for cardiac repair [7-9].

**Fig. (1):**
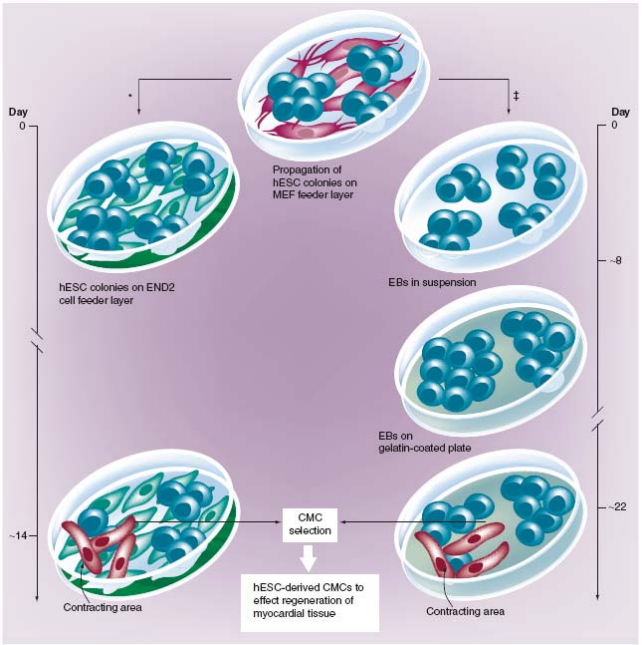
hESC propagation and *in vitro* differentiation into CMCs. hESC lines can be propagated continuously in the undifferentiated state when grown on top of an MEF feeder layer. With the Kehat protocol‡ [5], when hESCs are removed from these conditions and grown in suspension, they begin to generate three-dimensional differentiating cell aggregates termed embryoid bodies (EBs). Two weeks after plating on gelatin coated plates, spontaneously contracting areas appear within the EBs. The Mummery protocol* [6], however, uses END-2 cells in the place of MEFs as feeders for hESCs; within 2 weeks, spontaneously contracting areas appear in the hESC-colonies. (hESCs: human Embryonic Stem Cells; MEFs: Mouse Embryo Fibroblasts; EBs: Embryoid Bodies; END2: visceral endoderm-like cells; CMCs: cardiomyocytes)

Given the versatility of hESCs and the possibility of obtaining beating CMCs from them, (Fig. **1**) [6,10] they appear as the main candidate for cell-based applications for cardiac repair. In fact, hESCs apparently fulfill most, if not all, the properties of an ideal donor cell line [11] (Table **1**). 

In the following sections, we will discuss briefly, but critically, the obstacles on the path to hESC-based cardiac therapy. A possible strategy for cell-replacement therapy would be to initially allow spontaneous differentiation of ESCs into multiple lineages *in vitro* followed by selective purification of the cardiomyogenic lineage isolated from embryoid bodies (Fig. **1**). On this issue, Kehat *et al.* [5] show that transplanted hESC-derived CMCs substitute damaged pacemaker cells in a swine model of atrioventricular block, and are responsible for eliciting an ectopic rhythm compatible with the animal’s survival. Their results provide compelling evidence that this type of graft integrates electromechanically within the recipient tissue, as discussed extensively by Menaschè [12]. 

However, this is a relatively inefficient and haphazard process. We have to highlight that research on the exploitation of hESCs for cell-replacement therapy is still in its infancy, but the complex technical/technological problems are well worth overcoming when contemplating the benefits that this procedure may bring. Promising data has been obtained so far; hESC-based cell therapy will revolutionize medicine in the near future, offering therapeutical alternatives for treatment of severe degenerative disorders.

In point of fact, several obstacles still remain unsolved:

1. The yield of CMC production has to be dramatically improved. It is fundamental to work on the “ideal” culture conditions for CMCs differentiation. Unfortunately, the definition of strategies useful to the aim is not easy. The inherent differences between hESCs and their murine counterpart [5,12,13] necessitate the obligatory use of hESCs as a model; laws and ethical considerations place strong limitations to what can be done. A further complication is represented by differences between the various hESC lines [14-17] and their characterization which, to date, has been unsystematic. It appears that each hESC line possesses a unique expression signature and distinct cardiomyogenic inclination [18]. Hence, it is probably unrealistic to assume that an approach designed to improve cardiac differentiation would be applicable to all hESC lines. Clearly, systematic characterization is necessary to identify sub-categories of hESC lines. As underlined by Murdoch and co-workers [19], one possible solution to this problem is the establishment of national or international hESC banks which would allow comparable and detailed characterization of deposited cells and provide scientists with all necessary information to choose the most suitable hESC line for their own research.
2. Stimuli useful for directing hESC through the cardiac lineage are still only being investigated [20-23] A methodic, combinatorial approach, using various stimuli (trans-stimuli, extra-cellular matrices, co-culture, physical stimuli etc) could be the best way of directing the differentiation of stem cells *in vitro* in a cardiac stringent specific way. This speculation is supported by the fact that when in their natural milieu, cardiomyogenic differentiation of stem cells probably involves multiple signaling pathways. This may be mimicked *in vitro* with a combination of various methods that achieve a synergistic effect. In fact, *in vitro* derived prevascularized scaffold-free cardiac tissue patches from co-culture of cardiomyocytes, endothelial cells and fibroblasts were found to greatly improve cell viability post transplantation [24].
3. Culture media. For clinical applications, it is imperative to develop well-defined and efficient *in vitro* protocols for cardiomyogenic differentiation of stem cells, that utilize chemically defined culture media supplemented with recombinant cytokines and growth factors. The main drawback of the actual xenosupport system is the risk of cross-transfer of animal pathogens that might hamper future clinical applications. It was recently shown that non-human sialic acid Neu5Gc, against which many humans have circulating antibodies, is incorporated into hES cells grown on mouse feeder layers [25]. The use of human plasma-derived serum [26] and development of a serum-free support system [27] and animal-free feeder layer consisting of human fetal fibroblasts and adult epithelial cells [17] or foreskin cells [28] may provide an appropriate solution to these risks. Nevertheless, *in vitro* up-scaling of clinical grade cell products essentially free of xenogenic products in compliance with good manufacturing practice (GMP) remains a significant hurdle [29]. Universal acceptable solutions to these challenges are needed to provide the stringent levels of safety and quality control that would make the clinical applications of stem cell transplantation therapy realizable. Hopefully, this will be achieved in the near future.
4. Competency of derived CMCs in terms of excitation-contraction coupling. Another important issue is to what extent these cells can be considered mature CMCs in terms of excitation-contraction coupling. Indeed, heterogenous electrophysiological properties have been demonstrated in CMCs derived from separate differentiation methods within the same group [30]. This question cannot be accurately answered at the moment since the differentiation procedure has not been efficiently or even minimally standardized. However, some data [5] provide fairly convincing evidence that hESCs can integrate electrically with the recipient myocardium, suggesting that they are capable of contributing to the augmentation of pump function following injury.
5. Immune rejection has to be blocked. Upon differentiation, ES cells express molecules of the major histocompatibility complex (MHC), in particular MHC I, while MHC II expression levels are low or absent [31]. Thus, decreasing the expression of MHC I by genetic modification could improve immunologic tolerance. Alternatively, minimal but targeted conditioning of CD4 and CD8 T-cells may be an option to promote tolerance of embryonic stem cell-derived tissues [32].

On this issue, recent high-profile reports of the derivation of human embryonic stem cells from human blastocysts produced by somatic cell nuclear transfer (SCNT) [1,33,34] have highlighted the possibility of making autologous cell lines specific to individual patients. Given the range of immunophenotypes of hESC lines currently available, rejection of the differentiated cells by the host is a potentially serious problem. SCNT offers a means of circumventing this by producing embryonic stem cells of the same genotype as the donor. However, this technique is not without problems since it requires resetting of the gene expression program of a somatic cell to a state consistent with embryonic development [35].

Currently, the use of SCNT is under investigation from several points of view (ethical, scientific, technical/technological) and has a promising potential for treatment of a variety of degenerative diseases. Furthermore, with the advent of other techniques such as xeno-free [36,37] and direct differentiation of resident cells to cardiomyoyctes [38] may offer additional and exciting avenues for autologous cell therapy in the future.

6. Tumorigenicity may be a problem, even when terminally differentiated CMCs are used for cell replacement. Implantation of undifferentiated ES cells leads to the formation of benign teratomas in the recipients [34,35,39,40]

As discussed by Authors [41,42], an ES-derived teratoma is not in essence malignant, but its natural propensity to grow makes it potentially dangerous when implanted into an individual and, as such, a crippling obstacle on the path to ES cell therapeutics. Recent experiments suggest that formation of a teratoma may be dependent upon experimental conditions. Bjorklund *et al.* [43] have, for instance, shown that teratoma formation could be prevented in a majority of cases when pre-differentiated mouse ES cells were implanted into the rat brain at a very low density. Asano *et al.* [44] showed that ES cells implanted allogenically into a non-human primate fetus in utero formed a teratoma when developing in a natural cavity, but conversely integrated normally in tissues when implanted within various organs. Teratoma formation does not appear, therefore, as an unavoidable consequence of ES cell implantation but rather as a phenomenon, the mechanisms of which require further investigation in order to identify the safest procedures for clinical application. Tumorigenicity demands the use of an extensively characterized, pure, differentiated cell population. Negative selection of Oct4 (undifferentiation marker) expressing cells might be a solution. New strategies and methodologies need to be developed to isolate the terminally differentiated cells. ES cell implants can be tagged with some kind of death signal in such a way that when they start to form tumors, or cause severe complications, they can be cleared from the body, leaving the host unaffected. Other safeguards proposed to purify cardiomyocytes such as flow cytometry cell sorting using cardiomyocyte-specific fluorescent dye [45] or cardiac plasma membrane surface marker [46] and other strategies reviewed elsewhere [47] would further enhance the safety profile of these exogenously derived cardiomyocytes. As yet, there is no validated solution to this problem. 

## CO-TRANSPLANTATION IN REPAIRING MYOCARDIAL DAMAGE

Most studies with cell transplantation have been performed in animal models and patients with ischemic cardiomyopathy. Although results are promising, the most appropriate cell for this therapy is still a matter of discussion. Skeletal myoblasts transplantation has been shown effective in experimental [48-52] and clinical [53,54] studies. They differentiate into viable muscle fibers within the scared tissue but they lack morphological differentiation into cardiomyocytes and no intercalated discs develop between transplanted cells and the native adult cardiomyocytes. On the other hand, adult stem cells are pluripotent [55], but some studies suggested of only an angiogenic potential [56]. In the same model of ischemic cardiomyopathy, but comparing the effects between both cells separately we found that skeletal myoblasts transplantation resulted in myogenesis and improvement of ventricular function. In contrast, treatment with mesenchymal stem cells resulted in neoangiogenesis and no functional effect [57].

Manasché *et al* [58] demonstrated in a phase I clinical trial that skeletal myoblasts alone are able to improve ventricular function but with a high incidence of ventricular arrhythmias. One of the possible explanations is that when only new muscular fibres are provided (myoblast transplantation) these structures can become ischemic by the lack of vascularization and thus the tissue become more prone to arrhythmias. As some authors suggest that bone marrow stem cells have only an angiogenic potential [57] in a fibrosis, we have hypothesized that some problems could be eliminated providing contractile and angiogenic cells. The option for combined trasplantation of skeletal myoblasts and mesenchymal stem cells was based on pathophysiology of ischemic cardiomyopathy, characterized by chronic fibrosis and no vascularization of this region. This is the rationale for our studies with myoblasts and mesenchymal cells combined to get angiomuscular regeneration.

**Fig. (2):**
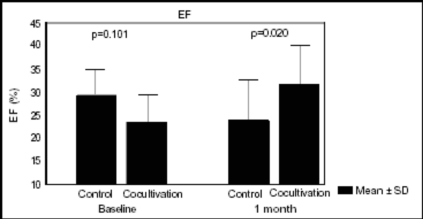
Ejection Fraction(EF%) of left ventricle between two groups and in the two periods of evaluation.

**Fig. (3):**
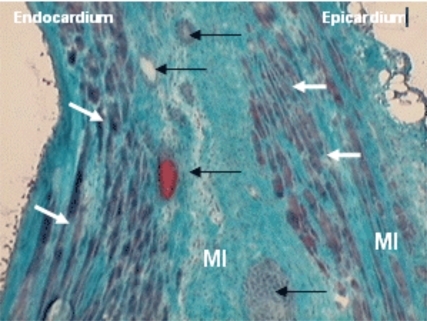
New skeletal fibers (white arrows) and new vessels and endothelial cells (black arrows) identified in a myocardial infarction (MI) (Gomery’s Trichrome, x 200).

We performed one study [59] in a model of myocardial infarction that observed increased ejection fraction after 30 days of both cells transplantation (myoblast and mesenchymal stem cells together) (24.03±8.68% to 31.77±9.06% p=0.011) and the difference was significant when this group was compared to control group at the same time. (31.77±9.06% vs 23.54±6.51% p=0.020) (Fig. **2**). Histological evaluation was made by Gomori’s Trichrome and identified cells with morphological characteristics of skeletal muscular fibers that colonize the region of fibrosis. The formation of new blood vessels was also identified in this region however, the presence of neither muscle nor blood vessels was visualized in the region of myocardial fibrosis in control group (Fig. **3**).

**Fig. (4):**
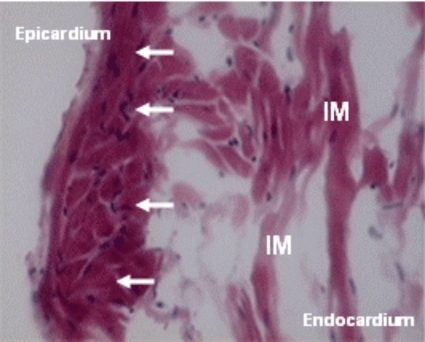
New skeletal fibers(white arrows) identified in an injured myocardium (IM) of Chagas disease. (H&E, X200).

Whether these same effects can be seen in other cardiomyopathies is still not known, so we performed one study on model of Chagasic cardiomyopathy [60]. We emphasize that in both described studies: Chagasic cardiomyopathy and myocardial infarction the co-transplantations included both cellular types co-cultured to allow *in vitro* interaction as reported previously [61]. The option for combined transplantation of skeletal myoblasts and mesenchymal bone marrow cells was based on pathophysiology of chagasic cardiomyopathy, characterized by chronic inflammation, sites of fibrosis and subendocardial ischemia. Cell transplantation in Chagasic model increased ejection fraction, reduced left ventricle volumes, both end systolic and diastolic (Table **2**). Histological evaluation was made by hematoxylin eosin and identified cells with morphological characteristics of skeletal muscular fibers that colonize the injured myocardium (Fig. **4**).

This effect on ventricular remodelling seems to be more related to the pathology and the way cells are transplanted into the heart than to the transplanted cell. Nevertheless, transplantation of co-cultured myoblasts and mesenchymal stem cells to a rat model of post-infarction ventricular dysfunction didn’t prevent ventricular remodelling despite of improvement in ventricular function [59]. Cells were injected only in anterior wall, differently from the current model where cells were injected in a more diffuse area (anterior and lateral wall). Further studies are necessary to evaluate whether the effect on ventricular remodelling is dependent on the model of cardiomyopathy and the way cells are deployed.

## FUNCTIONAL COMPARISON BETWEEN MONONUCLEAR AND MESENCHYMAL STEM CELLS IN A MODEL OF MYOCARDIUM INFARCTION

Experimental studies suggest that cardiac bone marrow stem cells (SC) transplantation can has a favorable impact on tissue perfusion. The strategy to repair the myocardial scar after infarction with SC has been proposed based on its capacity to differentiate according to the environment and could give a new perspective for myocardial regeneration [62][55,62-64]. Studies in human beings have shown improvement in clinical and functional cardiac status explained mainly by the angiogenic potential after stem cells transplantation [65-67]. Bone marrow stems cells are composed by mononuclear stem cells (MoSC) that contain hematopoietic stem cells with precursors of endothelial cells and mesenchymal stem cells (MeSC). MeSC shows pluripotentiality for mesoderm-derived cells, but it is only 0,01- 0,05% of MoSC although they can be enriched based on the adhesion capacity of MeSC on tissue culture plate [67]. To compare the functional outcome of mononuclear stem cells (MoSC) and mesenchymal stem cell (MeSC) therapy after myocardial infarction in rats, we used several approaches. The flow cytometric analysis we used was CD45+ and CD 34+ for the MoSC and CD45- and CD34- for the MeSC. The MeSC was positive for vimentin *in vitro*. There was difference in baseline LVEF and LVEDV between all groups. After one month, LVEF decreased in the control group and remained unchanged in MoSC and MeSC groups. The myocardium was remodeled in all of the groups (Tables **3** and **4**). Thus functional effectiveness was not demonstrated with both cell type therapy: MoSC and MeSC, when LVEF was analyzed by echocardiogram in this post-infarction dysfunctional model. Although we observed the stabilization of the MeSC therapy and no effects have been demonstrated to affect the ventricular dilatation. In spite of the fact that there was no improvement of the functional effects, the potential myocardial perfusion may warrant further analysis [68].

## HISTOPATHOLOGICAL COMPARISON BETWEEN MONONUCLEAR AND MESENCHYMAL STEM CELLS IN A MYOCARDIAL INFARCTION MODEL

**Fig. (5):**
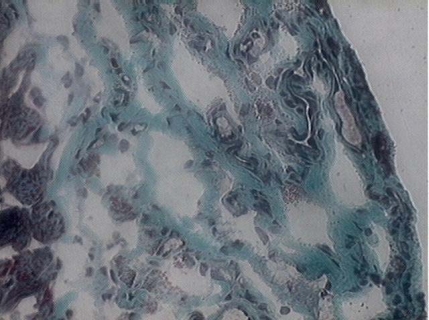
Angiogenesis: Multiple new vessels after MoSC therapy in the myocardial scar stained by Gomori's Trichrome (x 200).

**Fig. (6):**
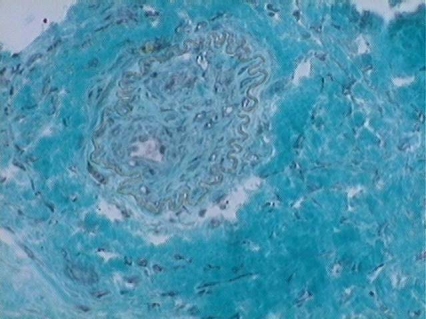
New vessel after MeSC in the myocardial scar stained by Gomori's Trichrome (x 400).

The histopathological analysis demonstrated new vessels in both cell type therapy, but with morphological differences. The new vessel formed in the scar after MoSC transplant is constituted of endothelial cells and lumen (Fig. **5**), while in the new vessels formed after the MeSC transplant (Fig. **6**), there was also the presence of smooth muscle. The latter was demonstrated by the presence of muscle in new vessel stained by anti-desmin with immunoflurescence in the myocardial scar. The control demonstrated only the scar with collagen deposit: fibrosis. This new vessel resulted from MeSC transplantation could have more physiological potential for vasomotor response, but the potential effect on myocardial perfusion may have great significance in cardiac recovery. Thus both cell type therapies: MoSC and MeSC have angiogenic capacity. The new vessel formed by MeSC transplant from the histopathological point of view, was complete and characterized for the presence of smooth muscle with endothelial cells and lumen [69]. Additional paracrine effects of stem and progenitor cells in cardiac repair has been extensively reviewed [70]. 

## CLINICAL USE OF MYOBLAST TRANSPLANTATION: RATIONALE

**Fig. (7):**
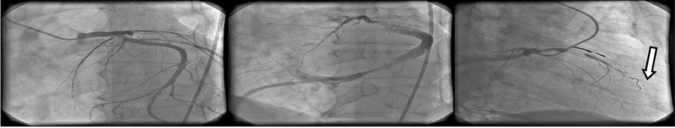
Procedural steps of cell transplantation using trans-coronary venous approach. Left: coronary artery visualization in LAO 30 view; middle: administration of the contrast medium via a guiding catheter placed in the coronary sinus in LAO 30 view, note the balloon inflated at the tip of the guiding catheter to slow the contrast medium outflow; right: placement of TransAccess® catheter system in the anterior interventricular vein and injection of the cell suspension via the IntraLume® microcatheter into anterior wall myocardium – the arrow indicates the microcatheter tip.

**Fig. (8):**
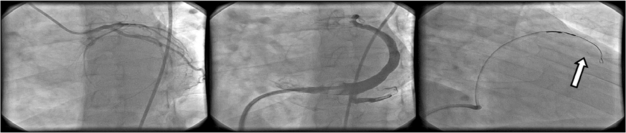
Procedural steps of cell transplantation using trans-coronary venous approach. Left: coronary artery visualization in LAO 30 view; middle: administration of the contrast medium via a non-occlusive guiding catheter placed in the coronary sinus; right: placement of TransAccess® catheter system in the anterior interventricular vein and injection of the cell suspension via the IntraLume® microcatheter into the septum – the arrow indicates the microcatheter tip.

**Fig. (9):**
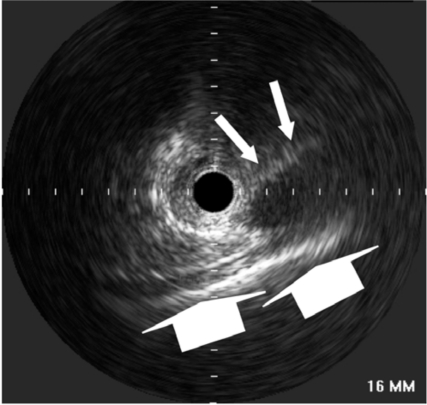
Intravascular ultrasound image obtained from the cardiac venous site. Please note the visibility of the pericardium (big arrows) and the coronary artery parallel to the vein (small arrows), enabling the orientation of TransAccess® catheter system.

Congestive heart failure caused by myocardial infarction continues to be a major clinical problem despite recent therapeutic advances. Formation of a fibrotic scar that replaces viable myocardium leads to decreased systolic function, left ventricular remodeling, aneurysm formation and subsequently to the progression of congestive heart failure. Unfortunately, cardiac transplantation may be an option only in selected end-stage heart failure patients, mainly due to organ shortage. Restoration of the total amount of contractile cells within the necrotic tissue as a result of cell transplantation into myocardium have been widely studied in both experimental and clinical conditions [71] Among variety of cell studied, autologous skeletal myoblasts, chronologically the first to enter the clinical arena, are one of the most encouraging cell source for cardiac repair because of their biologic properties and lack of ethical and immunological issues (Figs **7-9**). Transplantation of totipotent stem cell types, including bone marrow derived stem cells, into a fibrous postinfarction scar may result in their differentiation into fibroblasts [71-73], therefore direct myocyte precursors, myoblasts, have been considered as possible cell sources in patients with chronic, postinfarction myocardial injury.

Numerous studies have explored different delivery techniques. As skeletal myoblasts do not extravasate, their potential application in myocardial regeneration requires direct cell injection into the area of damaged myocardium. Therefore surgical approach (open-chest surgery) and several catheter-based methods had been proposed. 

## MYOBLAST TRANSPLANTATION DURING CARDIAC SURGERY

The initial clinical experience with autologous skeletal myoblast transplantation during open chest cardiac surgery was obtained by Ph. Menasché et.al., followed by phase one clinical trials performed both in Paris and in Poznan [58,74,75]. Both trials were done in patients in whom direct intramyocardial injections could be performed during coronary artery by-pass grafting (CABG). In the first clinical case described [58] several injections of the cell suspension into area of postinfarction injury within inferior wall of the left ventricle were performed. Five months after the procedure, a significant clinical status improvement was observed, including decrease of symptoms of heart failure by one NYHA class, an increase in segmental contractility and ejection fraction seen on echocardiography as well as increase in tracer activity on positron emission tomography suggesting new onset metabolic activity in the previously non viable scar area [58]. An independent phase one clinical trial on autologous skeletal myoblast transplantation in patients undergoing CABG [71,74,75] was performed in Poznan. MI survivors scheduled for CABG, with an akinetic area of the left ventricle and no viable myocardium was detected by means of dobutamine stress echocardiography were included into the study. A skeletal myocardial biopsy was obtained in all patients from the vastus lateralis. The biopsy sample was digested and myoblasts (satellite cells) were isolated. The cells were cultured for three weeks to increase the number of cells to be implanted. A measurable increase in segmental contractility was seen in all patients 2 to 3 months after the procedure and this effect was maintained throughout a 36-months follow-up period. However, 3 years after combined myoblast transplantation and CABG, in almost every third case in the Poznan series, end diastolic left ventricular diameter increased, suggesting that cell transplantation did not prevented left ventricular remodeling or the number of cells transplanted was not sufficient to prevent left ventricular dilatation.

A US-based multicenter phase one clinical trial aimed at evaluation of myoblast transplantation during CABG has also been reported [76]. Eleven patients underwent myoblast transplantation combined with CABG. Echocardiography, PET and MRI scans showed evidence of increased viability in the area of grafted scar. Improvement of the mean ejection fraction from 22.7% to 35.9% has been observed. Another phase one study evaluating myoblast injections during CABG has been reported by Herreros *et al.* [77], suggesting safety and feasibility of the method as well as indicating its possible efficacy in increasing contractile left ventricular performance.

Cell transplantations during cardiac surgery has certain advantages, including easy access and good visualization of the target site as well as possible delivery of big amount of cells per unit area. However, direct transepicardial approach may cause additional risk related to the surgery. It must be underlined that possible candidates for cell transplantation usually have a history of multiple infarctions, with significant LV dysfunction and clinical symptoms of severe heart failure, being high-risk candidates for open-chest surgery. Furthermore open-chest approach does not give free access to septal wall which is frequently inflicted by postinfarction injury. 

Clear interpretation of clinical data obtained from trials evaluating cell transplantation during CABG is not possible. The effect of CABG and cellular transplantation performed at the same time cannot be easily distinguished. Despite the use of careful inclusion criteria aimed at selection of patients with no viable myocardium within the target postinfarction area, the possible effect of skeletal myoblast transplantation may be enhanced by myocardial revascularization. Large on-going and future clinical trials evaluating efficacy of myoblast injections during CABG may allow evaluating of the cell effect independently on restoration of blood flow. Nevertheless, a recently concluded randomized, placebo-controlled study of 97 patients, the myoblast autologous grafting in ischemic cardiomyopahty (MAGIC) trial failed to show improved LV function at 6 months after myoblast transplantation [78]. 

## PERCUTANEOUS MYOBLAST TRANSPLANTATION

Myoblast transplantations, performed as a sole therapy during percutaneous procedures, may allow the evaluation of the cellular effect independent of revascularization. In addition, it may enable repeated injections in patients with severe myocardial injury, since excessive numbers of transplanted cells in a single injection may result in a small percentage of survived and grafted cells. Initial experience has been obtained with cell injection into myocardium with the use of both endoventricular catheter systems [79] and transvenous approach [80,81]. 

The efficacy of cell transplantation may be very much dependent on the design of catheter system used [82,83]. Currently available endoventricular catheter systems, utilized for intramyocardial injections of therapeutic agents, have limited stability, since the injection pressure can cause expulsion of the needle tip from the injection site. After needle withdrawal from a short perpendicular injection channel, therapeutic agent leakage back to the ventricle may occur. Such a back-flush of cells from the puncture site may cause the presence of graft cells in the systemic circulation and/or diminished number of cell delivered to the target area. Moreover, for endoventricular systems in which needle is directed perpendicularly to the inner surface of cardiac muscle wall and does not follow the heart movement, thinned postinfarction scar is currently a relative contraindication. 

Another catheter-based endovascular system for direct myocardial injection using IVUS guided needle punctures via the coronary venous system (the TransAccess®, Trans Vascular, Manlo Park, CA) has been recently developed [84,85]. The TransAccess® catheter is a monorail, composite catheter system combining both a phased-array IVUS and a pre-shaped adjustable nitinol needle. After placing the TransAccess® system in the target coronary vein, through the coronary sinus, the system is oriented using the corresponding artery, pericardium and ventricular myocardium as landmarks with IVUS imaging. The nitinol needle is then extended into the myocardium and a microinfusion catheter (IntraLume™, TransVascular Inc.) is then advanced through the needle deep into the myocardium with simultaneous injection of the therapeutic agent. 

The POZNAN trial [81] was performed as a phase one clinical trial for both the TransAccess® catheter system and for percutaneous autologous myoblast transplantations performed as a sole therapy. The trial has confirmed the feasibility of intramyocardial injections performed using the TransAccess® system. Precise advancement of the micro lumen catheter in the remote target area up to 4 cm deep within the injured myocardium was obtained. The use of both the anterior interventricular vein as well as the middle cardiac vein, parallel to the posterior descending coronary artery, has been shown to be feasible. In the POZNAN trial, in 4 cases the middle vein was used to advance the TransAccess® system closer to the apical segments of the left ventricle, as compared to cases using the anterior interventricular venous approach [81]. The lack of procedural success in one patient, related to the inability of appropriate positioning of the guiding catheter across the venous valve present at the bifurcation of the great cardiac vein, suggests the need for better coronary sinus guiding catheter design (10F guiding catheters were used). In remaining POZNAN trial patients, two to four intramyocardial injections 1.5-4.5 cm deep were performed in each patient, delivering up to 100 million cells in 0.6 – 2.5 ml of saline. During 6 months follow-up NYHA class improved in all patients and ejection fraction assessed by echocardiography increased 3-8 percent in 6 out of 9 cases [81,83]. 

Smits *et al.* [79] used transventricular approach to inject myoblast suspensions into the area of postinfarction injury. They observed increase in LV ejection fraction assessed by angiography at 3 month follow-up, but nucluear radiography and magnetic resonance failed to confirm this improvement. However, they recently published a very interesting study [86] have shown hemodynamic improvement evaluated by pressure-volume loops after follow-up for up to one year after percutaneous myoblast transplantation. 10-15 injections of autologous myoblasts using Myostar™ (Cordis, Warren, NJ) resulted in increased ejection fraction at 6 months, increased cardiac output, reduction of end-systolic volume and a trend towards improved stroke work. Another recently published study [86] decribes results of transventricular injections performed using fluoroscopy guided MyoCath™ catheter (Bioheart, Weston, FL) or the NOGA™-guided catheter system (Biosense-Webster, Waterloo, Belgium). The study failed to show improvement in the ejection fraction but wall motion score index improved both at rest at under low-dose dobutamine. In addition, the result of the study indicate the possibility of arrhythmogenic effect of myoblast transplantation.

## MYOBLAST-RELATED SAFETY ISSUES

Myocardial and skeletal muscle tissues differ significantly in their electromechanical properties. Cardiac cells, having special cell-cell junctions, even though separated one from another, act together synchronically. Junctions contain adherins and gap junctions for mechanical and electrical coupling. Cardiac tissue gap junctions contain connexin-43 transmembrane protein by which electrical current can be fast and freely conducted [3]. Although certain data suggest that skeletal myoblasts may acquire few characteristics of cardiomyocytes, generally it could be assumed that the grafted cells do not transdifferentiate and keep morphological and electrophysiological properties of skeletal muscle [87,88]. It is speculated that skeletal myoblasts (satellite cells) are not able to form intercellular junctions characteristic for cardiomyocytes. However, it was shown that the lack of junctions between grafted cells and host tissue do not preclude improvement in LV contractile function [87].

There is evidence that this positive effect of skeletal myoblasts on myocardial contractility seems to last over time and is correlated with the number of implanted cells [50]. Experimental studies performed on myocardial wound strips indicate that skeletal myoblast grafts do contract when exogenously stimulated [49]. In addition, cardiomyocytes and skeletal myoblasts, when placed in co-culture, forms synchronous beating network [89]. It has been suggested that transplanted cells can contract synchronously even in the absence of connections between cells, because a simple stretch may initiate contraction [90]. This phenomenon is important due to the possibility of insulation of transplanted cells by scar tissue. The scar may form a physical barrier which impede electromechanical coupling. 

It is speculated that the inability of skeletal myoblasts to transdifferentiate to cardiomyocytes and to form junctions with neighboring cells may be a substrate for ventricular re-entry arrhythmia. Indeed, current experimental and clinical data suggest a possibility of increased risk of arrhythmogenicity. In their first clinical series, Menasché at al. [58] implanted automatic internal cardioverter-defibrillators (AICD) in 4 out of 10 patients receiving autologous skeletal myoblast transplantations during CABG due to sustained episodes of ventricular tachycardia (VT). The possible arrhythmogenic effect have been also noticed in an endoventricular catheter-based trial which had to be temporarily stopped because of 2 sudden deaths, probably due to arrhythmia. In the Poznan CABG phase one experience [74,75], episodes of sustained ventricular tachycardia (VT) were observed in first 2 patients during early postoperative period, but prophylactic amiodarone administration in other patients prevented VT episodes and no amiodarone treatment was continued later then 6 weeks during follow-up. This corresponds to the experience of Menasche *et al* [58] Later during follow-up period only one of their four AICD patients experienced asymptomatic VT episodes [71]. On the other hand, observations from percutaneous series in the POZNAN trial, indicating successful prevention of cell transplantation-related ventricular arrhythmia by prophylactic amiodarone administration, suggest that AICD implantations may not necessarily needed in all patients that undergo myoblast transplantations [81,83]. 

Based on published data from clinical studies, it could be speculated that the possible arrhythmogenic effect of myoblast transplantation is noticed only in the initial weeks after the procedure. The possible arrhythmogenic effect of myoblast transplantation is more probably related to its mechanics, including myocardial puncture and the inflammatory response to transplanted cells, some of which die after injection, than to possible problems with electromechanical coupling between newly developed myocytes and cardiomyocytes. Possible electromechanical coupling problems would result in late arrhythmia as cells differentiate (downregulation of connexin-43 and N-cadherin), a situation that has not been observed in clinical trials so far. 

At the current stage, only moderate numbers of patients that have undergone autologous skeletal myoblast transplantations, it is difficult to predict whether skeletal myoblasts are really arrhythmogenic, especially that patients with ischemic LV dysfunction frequently develop ventricular arrhythmia. Indeed, the experience of MAGIC trial [78] reflected this trend whereby no significant difference in ventricular arrhythmias or major cardiac adverse events was detected between myoblast-treated patients and placebo control group at 6-month follow-up. Nevertheless, future studies on cell transplantation in patients with postinfarction heart failure will have to focus on potential arrhythmogenic effect. Similarly, large phase two/three clinical trials are needed to assess the efficacy of myoblast transplantation in chronic postinfarction myocardial injury.

## Figures and Tables

**Table 1.** hESCs Meeting the Need for Cell-based Applications for Cardiac Repair (hESC:human Embryonic Stem Cell; CMC Cardiomyocytes; MHC:Major Histocompatibility Complex)

Ideal donor cell line properties	hESC line properties
Electrophysiological, structural and contractile properties comparable to CMCs; ability to inegrate structurally and functionally with host tissue	Plurality of cell lineage for transplantation of specialized CMC subtypes (pacemaking, atrial, ventricular, etc.) and/or endothelial progenitor cells to induce angioenesis
Retain an initial proliferative potential for colonization of the scar tissue	*In vitro*, differentiate schemes give the opportunity to choose the ESC-maturation stage
Ability to undergo genetic manipulation *ex vivo* to promote desirable characteristics (i.e. minimal immunogenicity; resistance to ischemia, apoptosis or antibiotics )	Clonal origin gives the opportunity for extensive characterization and genetic manipulation (e.g. decrease the expression of MHC, to preform an antibiotic-cell selection *in vitro*
Autologus origin	Derivation of ESC lines specifically for each patient with somatic nuclear transfer
Large quantities for transplantation	Currently the only cell source with this potential property

**Table 2.** Functional Evaluation of Cells Transplantation in a Model of Chagas Disease

Group	Control	Co-culture	Difference Between Groups (p)
LVEDV. ml pre	0.69±0.11	0.82±0.07	
LVEDV. ml post	0.73±0.14	0.65±0.14	0.0166
P	0.6311	0.0004	
LVESV. ml pre	0.44±0.08	0.56±0.05	
LVESV. ml post	0.46±0.12	0.32±0.09	0.0001
P	0.6523	<0.0001	
EF% pre	36.74±3.63	30.10±5.71	
EF% post	37.42±6.66	51.76±6.63	<0.0001
P	0.7684	<0.0001	

**Table 3.** Baseline and After 1-month LVEF

Variable	Control (n=21)	MeSC (n=13)	MoSC (n=8)	p*
	Mean± SD	Mean± SD	Mean± SD	
LVEF baseline	26.84±7.05	26.62±7.34	21.79±8.77	0.2505
LVEF 1-month	22.32±6.94	25.55±10.21	18.60±6.11	0.2980
p⟡	0.0045	0.6505	0.4232	

(*) ANOVA

(**) Adjusted to baseline

(⟡) Paired t test (p<0.05)

LVEF (Left Ventricular Ejection Fraction).

**Table 4.** Differences Between Baseline and 1-month

Variable (1month-baseline)	Control (n=21)	BMSC (n=13)	Mononuclear (n=8)	p
	Mean±SD	Mean±SD	Mean±SD	
LVEDV	0.13±0.16	0.16±0.06	0.39±0.16	0.0025**
LVESV	0.13±0.13	0.13±0.09	0.34±0.13	0.0004*
LVEF	-4.53±6.48	-1.07±8.30	-3.19±10.61	0.4733*

(*) ANOVA

(**) Kruskal-Wallis (p<0.05)

LVEDV (Left Ventricular End Diastolic Volume), LVESV (Left Ventricular End Systolic Volume) and LVEF (Left Ventricular Ejection Fraction).
